# Seroprevalence and molecular diversity of Human Herpesvirus 8 among people living with HIV in Brazzaville, Congo

**DOI:** 10.1038/s41598-021-97070-4

**Published:** 2021-08-31

**Authors:** Gervillien Arnold Malonga, Aude Jary, Valentin Leducq, Dimitry Moudiongui Mboungou Malanda, Anicet Luc Magloire Boumba, Elodie Chicaud, Isabelle Malet, Vincent Calvez, Jean Felix Peko, Anne-Geneviève Marcelin

**Affiliations:** 1 Sorbonne Université, INSERM, Institut Pierre Louis d’Epidémiologie et de Santé Publique, Assistance Publique – Hôpitaux de Paris (AP-HP), Hôpitaux Universitaires Pitié-Salpêtrière - Charles Foix, Laboratoire de Virologie, Department of Virology – CERVI, Pitié-Salpêtrière Hospital, 83 boulevard de l’Hôpital, 75013 Paris, France; 2 Faculté des Sciences de la Santé, Université Marien Ngouabi, Brazzaville, Republic of Congo; 3 Service d’Anatomie et Cytologie Pathologiques, Centre Hospitalier Universitaire de Brazzaville, Brazzaville, Republic of Congo; 4 Laboratoire d’Analyses Médicales, Hôpital Général de Loandjili, Pointe-Noire, Republic of Congo

**Keywords:** Immunological techniques, DNA sequencing, Infection, Herpes virus, Oncogenes

## Abstract

Human herpesvirus 8 (HHV8) is endemic in Africa, although studies of this infection are rare in Congo. We evaluated seroprevalence and HHV-8 diversity among people living with HIV. We included 353 patients receiving highly active antiretroviral therapy. Antibodies against HHV-8 latency-associated nuclear antigen were detected by indirect immunofluorescence. In HHV-8 positive patients, we performed HHV-8 quantification in blood and saliva by real-time PCR and typing by Sanger sequencing of K1 open reading frame. HHV-8 seroprevalence was 19%, being male (odd ratio [OR] = 1.741, [95% Confidence interval {CI}, 0.97–3.07]; *p* = 0.0581) and having multiple sex partners before HIV diagnosis (OR = 1.682, [CI 95%, 0.97–2.92]; *p* = 0.0629) tended to be associated with HHV-8 seropositivity. Of the 64 HHV-8 seropositive patients, HHV-8 DNA was detected in 10 (16%) in saliva, 6 (9%) in whole-blood and in 2 (3%) in both whole-blood and saliva. Three out of 6 HHV-8 strains were subtypes A5, 2 subtype B1 and 1 subtype C. HHV-8 seroprevalence was relatively low with more frequent carriage in men, associated with asymptomatic oral excretion and a predominance of subtype A5. These data tend to support the hypothesis of horizontal transmission in people living with HIV in Brazzaville.

## Introduction

Human Herpesvirus 8 (HHV-8), also called Kaposi's sarcoma-associated herpesvirus (KSHV) was discovered in 1994 in the tissues of Kaposi's sarcoma (KS) AIDS patients^1^. It’s considered to be the causative agent of all forms of KS, including classic, endemic, iatrogenic and epidemic forms and is also associated with two hemopathies: primary effusion lymphoma (PEL) and multicentric Castleman's disease (MCD). The main pathway for HHV-8 infection is thought to be via salivary excretion, which explains the presence of large HHV-8 viral particles and high titters in saliva^2^. Epidemiology of KS is closely correlated with the seroprevalence of HHV-8. Globally, the seroprevalence of HHV-8 is very heterogeneous according to regions of the world and populations. Excluding the cohorts of HIV-infected patients, this seroprevalence falls into three groups: (i) in Northern Europe, Asia and North America, less than 10% of the general population has HHV-8 antibodies, (ii) Mediterranean region constitutes an intermediate zone with a seroprevalence of between 10 and 30%, (iii) in Sub-Saharan Africa, on the other hand, this is more than 50%; making it a highly endemic area for HHV-8 with a prevalence unevenly distributed^3–5^. HIV-infected population remains at an 800-fold elevated risk of KS compared with the general population^6^ , making it the most common cancer in people living with HIV (PLWH) in Sub-Saharan Africa^7^. Indeed also correlation with HIV status remains debated, the seroprevalence of HHV-8 infection is reported to be higher, both among HIV-positive women, heterosexual men and men who have sex with men^3,8^. Unlike other populations, in HIV patients, HHV-8 seroprevalence is very high regardless of area of residence, with high, medium or low incidence. Sub-Saharan Africa suffers the brunt of this co-infection, with seroprevalences for HHV-8 of 48% in South Africa, 51% in Zambia, 56% in Uganda reported in HIV patients^3^ ; 62%, 65.6% and 79% respectively in Nigeria, Ghana and Cameroon^9–11^. With an estimated HIV prevalence of 3.2% among 15 to 49 year olds, Congo-Brazzaville is also facing the problem of HIV and by extension HHV-8^12^.

The subtypes distribution of HHV-8 varies according to geography and ethnic origin. Molecular epidemiological analysis of the K1 open reading frame (ORF-K1) identified 7 subtypes of HHV-8 (A, B, C, D, E, F and Z). Subtypes A and C are found in Europe, North America, Middle East and North Asia^13–15^; subtypes B and A5 are characteristic of Africa^16^; subtype D, on Pacific Islands and in Taiwan^17^; subtype E, in Native Americans and Brazilians^18^; Subtype F, firstly identified in Uganda^19^ and recently described in France^20^, and subtype Z, in a small cohort of Zambian children^21^.

As it stands, no studies have been done on HHV-8/HIV coinfection in Congo-Brazzaville. Although the country is considered an endemic area for these two viruses, the seroprevalence and subtypes of HHV-8 circulating in people living with HIV remains unknown. We conducted this study to evaluated HHV-8 seroprevalence and associated risk factors among people living with HIV in Brazzaville. We also determine the viral load and subtype diversity of HHV-8 circulating in our study population.

## Results

### Patients’ characteristics

A total of 353 PLWH under HAART (Highly active antiretroviral therapy) in Brazzaville agreed to participate, from whom paired saliva-and-blood samples were obtained. Analysis of the sociodemographic characteristics of the participants (Table 1) indicated that 256 (73%) were female, mainly Congolese 345 (98%) and median age was 45 [IQR, 39–54] years. According to marital status, 163 (46%) were single, 70 (20%) married and 55 (16%) widowers, while the common-law union and the divorced were 43 (12%) and 22 (6%), respectively. Half (n = 178) had secondary level of education followed by primary level (n = 98, 28%). Almost 40% (n = 130) of patients had an HIV stage mentioned in the file at initiation, more than half of whom (n = 72, 55%) had stage 3 and 4, late. Median delay since start of HIV-treatment was 4 [1–9] years and median CD4 count 372.5 [230.3–552.3] cells/µl. Most PLWH were in first-line treatment 342 (97%). Sex workers, MSM and patients who have received a previous blood transfusion represented 14 (4%), 4 (4%) and 103 (29%), respectively. Majority of men were circumcised (n = 91, 94%) and very few were IDU (Intravenous drug users) 3 (1%). One hundred and sixty-three (46%) patients reported multiple sex partners before HIV diagnosis and 63 (18%) always used condoms *versus* 290 (82%) who only used them sometimes or never. Note that, 112 (32%) engaged in unsafe sex practices and only 41 (12%) reported a history of STIs.

**Table 1:** Epidemiological characteristics of study population.

Variable	General population of PLWH (N = 353)	HHV-8 positive (n = 64)	HHV-8negative (n = 277)	*P *value
	No. (%)	No. (%)	No. (%)	
Male	97 (27.48)	24 (37.50)	71 (25.63)	0.056
Female	256 (72.52)	40 (62.50)	206 (74.37)	
Congolese nationality	345 (97.73)	60 (93.75)	273 (98.56)	0.044
Other nationalities*	8 (2.27)	4 (6.25)	4 (1.44)	
Age, median (IQR), years	45 (39–54)	45 (36,25–54)	45 (39–55)	0.71
Single	163 (46.18)	28 (43.75)	129 (46.57)	0.4
Married	70 (19.83)	15 (23.44)	53 (19.13)	
Widower	55 (15.58)	6 (9.38)	47 (16.97)	
Common-law union	43 (12.18)	9 (14.06)	33 (11.91)	
Divorced	22 (6.23)	6 (9.38)	15 (5.42)	
Uneducated	8 (2.27)	1 (1.56)	7 (2.53)	0.1
Primary	98 (27.76)	14 (21.88)	78 (28.16)	
Secondary (I & II)	178 (50.42)	32 (50)	142 (51.26)	
Superior	69 (19.55)	17 (26.56)	50 (18.05)	
Early HIV (Stage 1 & 2) stage at initiation	58 (16.43)	13 (20.31)	43 (15.52)	NC**
Late HIV (Stage 3 & 4) stage at initiation	72 (20.40)	9 (14.06)	60 (21.66)	
Not specified stage at initiation	223 (63.17)	42 (65.63)	174 (62.82)	
Duration since treatment, median (IQR), y	4 (1–9)	4 (1–7)	4,5 (1–9)	0.12
CD4 count, median (IQR), Cell/µl	372.5 (230.3–552.3)	478.5 (256.5–613)	330.5 (220.3–515)	0.84
First line treatment	342 (96.88)	61 (95.31)	269 (97.11)	0.44
Second line treatment	11 (3.12)	3 (4.69)	8 (2.89)	
Non-medical personnel	343 (97.17)	62 (96.88)	270 (97.47)	0.68
Nurse	3 (0.85)	0	3 (1.08)	
Midwife	3 (0.85)	1 (1.56)	1 (0.36)	
Pharmacy vendor	2 (0.57)	0	2 (0.72)	
Red cross staff	1 (0.28)	1 (1.56)	0	
Laboratory technician	1 (0.28)	0	1 (0.36)	
Sex worker	14 (3.97)	5 (7.81)	9 (3.25)	0.15
MSM	4 (4.12)	1 (4.17)	2 (2.82)	˃ 0.99
Past blood transfusion	103 (29.18)	14 (21.88)	86 (31.05)	0.15
Circumcision	91 (93.81)	22 (91.67)	67 (94.37)	0.64
IDUs	3 (0.85)	1 (1.56)	2 (0.72)	0.46
History of STI(s)	41 (11.61)	10 (15.63)	31 (11.19)	0.33
Multiple sex partners before HIV diagnosis	163 (46.18)	36 (56.25)	120 (43.32)	0.07
Condom use	63 (17.85)	9 (14.06)	225 (81.23)	0.38
Unsafe sex practices	112 (31,73)	24 (37.50)	85 (30.69)	0.29
Surgical history	9 (2,55)	1 (1.56)	7 (2.53)	˃ 0.99

* Including Central African, Beninese, Cameroonian, DR Congolese, Senegalese.

** no data for everyone. Abbreviation: HIV: human immunodeficiency virus; IDUs: intravenous drug users; IQR: interquartile; MSM: men who have sex with men; NC: non calculated; PLWH: people living with HIV; STI: sexually transmitted infection.

### HHV-8 seroprevalence

In our study, 12 (3%) patients had equivocal HHV-8 serology results and were not included in our seroprevalence estimation. Characteristic of these patients are described in Table S1.

Overall, the seroprevalence of HHV-8 was 19% (n = 64). The presence of HHV-8 antibodies tended to be higher in males than females (25% *versus* 16%, *p* = *0.056*) and in patients with history of multiple partners before HIV infection (*p* = 0.07). However, no difference according to age, CD4 cells count, MSM or IDUs status was found (Table 1). None of the patients studied reported symptoms associated to HHV-8 related diseases.

### HHV-8 viral load and ORF-K1 phylogenetic analysis

Of the 64 patients with positive antibodies against LANA-1, 10 (16%) had HHV8-DNA oral shedding and 6 (9%) a detectable HHV8-DNA viral load in whole blood. Only 2 (3%) patients had detectable HHV-8 DNA viral load in both saliva and whole blood. The characteristics of the patients in whom the HHV-8 DNA was detectable are described in Table S2. Median HHV8-DNA viral load in oral shedders was 3 [IQR, 2.7 – 3.4] log_10_ copies/10^6^ cells (Table 2). Comparison between HHV8-DNA oral shedders and non-oral shedders showed no statistically significant difference (Table 3). It is noteworthy that all 12 individuals who had HHV-8 equivocal results, did not shed virus either in saliva or in whole blood.

**Table 2:** Description of HHV-8 characteristics in patients with detectable viral load.

Sample ID	Sex	Age (years)	Saliva viral load	Whole blood viral load	Subtype	GenBank accession numbers
			Log	Log		
HT-243	Female	42	2.95	Undetectable	A	MW892531
EE-138	Female	65	3.25	Undetectable	A	MW892532
MM-003	Male	30	3.57	Undetectable	A	MW892533
MM-024	Female	54	4.83	Undetectable	C	MW892534
CHI-125	Male	46	undetectable	2.57	B	MW892535
CT-282	Male	26	2.63	Undetectable	B	MW892536
HM-034	Female	30	2.68	0.35	NA	ˉ
CHH-099	Female	29	3.54	3.28	NA	ˉ
HT-178	Male	53	1.01	1.15	NA	ˉ
HT-204	Male	45	3.14	Undetectable	NA	ˉ
CT-295	Female	30	2.85	Undetectable	NA	ˉ
MM-015	Female	54	undetectable	2.04	NA	ˉ
CHI-094	Female	52	undetectable	1.36	NA	ˉ

NA: not amplifiable.

**Table 3:** Comparison between HHV-8 oral shedders and non-oral shedders.

Variable	HHV-8 oral shedding (N = 10)	Not HHV-8 oral shedding (N = 54)	*P *value
	No. (%)	No. (%)	
Male	4 (40)	20 (37)	> 0.99
Female	6 (60)	34 (63)	
Congolese nationality	10 (100)	50 (93)	> 0.99
Other nationality	0	4 (7)	
Age, median (IQR), years	37 (30–49)	46 (38–54)	0.11
Single	7 (70)	21 (39)	0.36
Married	2 (20)	13 (24)	
Widower	0	6 (11)	
Common-law union	1 (10)	8 (15)	
Divorced	0	6 (11)	
Uneducated	0	1 (2)	0.68
Primary	3 (30)	11 (20)	
Secondary (I & II)	6 (60)	26 (48)	
Superior	1 (10)	16 (30)	
Early HIV (Stage 1 & 2) stage at initiation	3 (30)	10 (19)	NC
Late HIV (Stage 3 & 4) stage at initiation	2 (20)	7 (13)	
Not specified stage at initiation	5 (50)	37 (68)	
CD4 count, median (IQR), Cell/µl	205 (187–391)	508 (398–625)	NC
First line treatment	9 (90)	52 (96)	0.40
Second line treatment	1 (10)	2 (4)	
Non-medical personnel	10 (100)	52 (96)	> 0.99
Medical personnel (Midwife, Red cross staff)	0	2 (4)	
Sex worker	0	5 (9)	> 0.99
MSM	0	1 (2)	> 0.99
Past blood transfusion	3 (30)	11 (20)	0.67
Circumcision	4 (100)	18 (33)	> 0.99
IDUs	0	1 (2)	> 0.99
History of STIs	2 (20)	8 (15)	0.64
Multiple sex partners before HIV diagnosis	4 (40)	32 (59)	0.31
Condom use	0	9 (17)	0.33
Unsafe sex practices	2 (20)	22 (41)	0.29
Surgical history	1(10)	0	> 0.99

HIV: human immunodeficiency virus; IDUs: intravenous drug users; IQR: interquartile; MSM: men who have sex with men; NC: non calculated; STI: sexually transmitted infection

**Figure 1:**
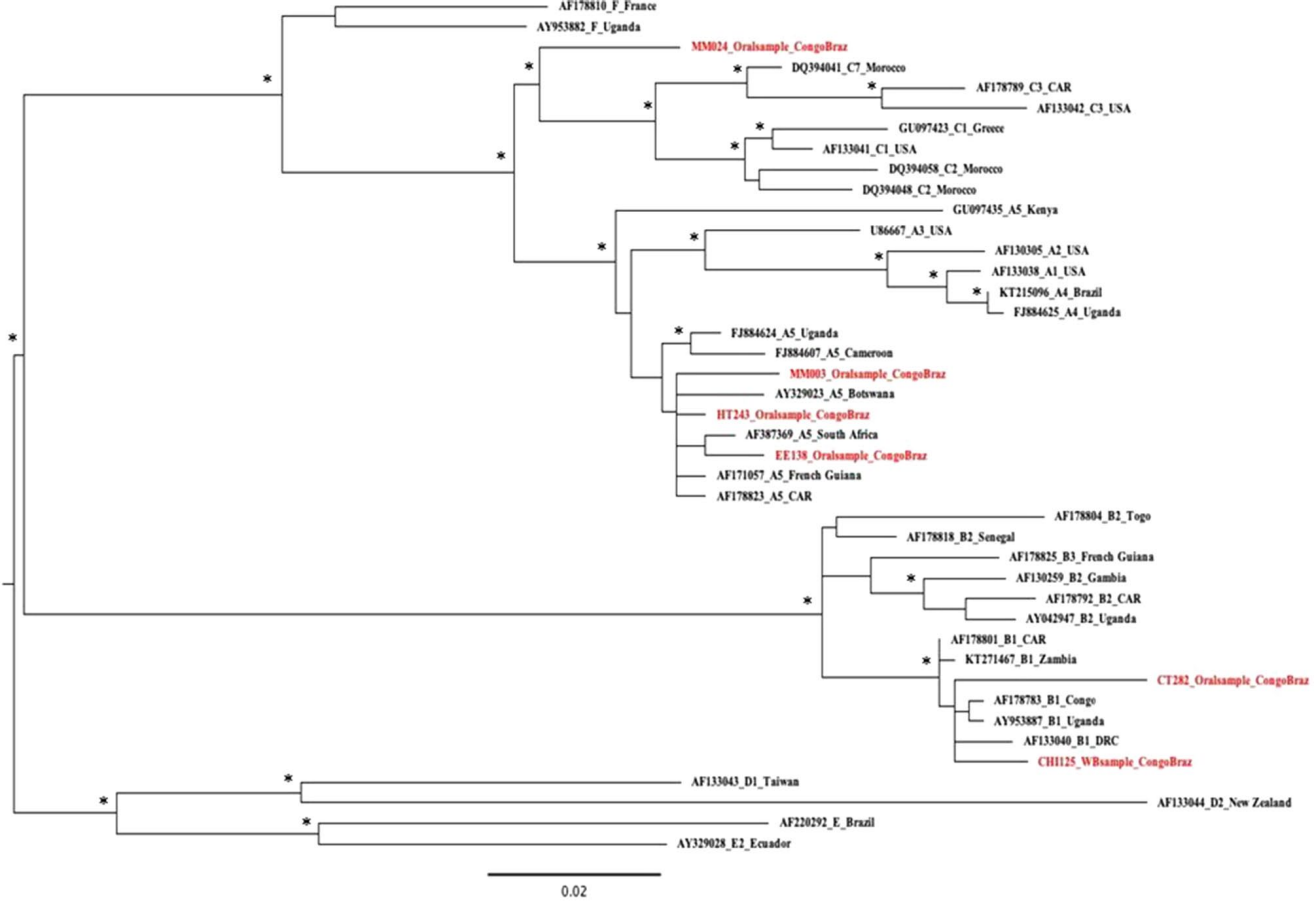
Maximum likelihood phylogenetic tree constructed with nucleotide ORF K1 sequences issued from patients with detectable HHV8-DNA viral load in saliva and whole blood sample. *Tree was constructed with PhyML software (version 3.0) with the GTR model, four rate categories of gamma shape parameter and 1000 bootstrap resampling and visualized in FigTree (version 1.4.3. Patients’ sequences are shown in red, reference sequences from NCBI database in black and asterisks represent nodes with a bootstrap support* > *70%. GenBank reference sequence accession numbers were as follow:* A1: AF133038 (USA), A2: AF130305 (USA), A3: U86667 (USA), A4: KT215096 (Brazil), A4: FJ884625 (Uganda), A5: AF178823 (CAR), A5: FJ884607 (Cameroon), A5: AY329023 (Botswana), A5: FJ884624 (Uganda), A5: GU097435 (Kenya), A5: AF387369 (South Africa), A5: AF171057 (French Guiana), B1: AF133040 (Democratic Republic of the Congo), B1:AF178783 (Republic of Congo), B1: AF178801 (CAR), B1: AY953887 (Uganda), B1: KT271467 (Zambia), B2: AF178792 (CAR), B2: AY042947 (Uganda), B2: AF130259 (Gambia), B2: AF178818 (Senegal), B2: AF178804 (Togo), B3: AF178825 (French Guiana), C1: GU097423 (Greece), C1: AF133041 (USA), C2: DQ394048 (Morocco), C2: DQ394058 (Morocco), C3: AF178789 (CAR), C3: AF133042 (USA), C7: DQ394041 (Morocco), D1: AF133043 (Taiwan), D2: AF133044 (New Zealand), E: AF220292 (Brazil), E2: AY329028 (Ecuador), F: AY953882 (Uganda), F : AF178810 (France). GenBank new sequence accession numbers were as follows: HT243_Oralsample_CongoBraz: MW892531; EE138_Oralsample_CongoBraz: MW892532; MM003_Oralsample_CongoBraz: MW892533; MM024_Oralsample_CongoBraz: MW892534; CHI125_WBsample_CongoBraz: MW892535; CT282_Oralsample_CongoBraz: MW892536.

ORF-K1 coding region amplification by nested PCR was successful for 6 of the 12 who had a detectable viral load and a phylogenetic tree based on the same region implemented with reference sequences was constructed (Fig. 1). Subtype A5 was the most common (n = 3; 50%), followed by B1 (n = 2; 33%). Finally, one patient was infected with subtype C (n = 1, 17%).

### Risk factors associated with HHV-8 seropositivity

Risk factors associated with HHV-8 seropositivity were investigated using logistic regression analysis; for a detailed analysis of the association between education level and HHV-8 seropositivity, subjects were divided into 2 categories: secondary/superior and uneducated/primary. On univariate analysis, being male (*odd ratio [OR]* = *1.741, [95% Confidence interval {CI}, 0.97—3.07]; p* = *0.0581*) and having multiple sex partners before HIV (*OR* = *1.682, [CI 95%, 0.97 – 2.92]; p* = *0.0629*) tended to be associated with HHV-8 seropositivity (Table 4). No association was observed with age, education level, sex workers, MSM, blood transfusion, circumcision, history of STIs, unprotected sex, intravenous drug users, history of surgery and CD4 count cells. In multivariate analysis, no particular pattern emerges between HHV-8 status and associated independent factors (Table 4).

**Table 4:** Risk factors associated with HHV-8 seropositivity.

Variable	Univariate results			Multivariate results		
	OR	95% CI	*P *value	OR	95% CI	*P *value
Sex	**1.74**	**0.97—3.07**	**0.05**	1.45	0.34—5.83	0.59
Age	0.99	0.97—1.01	0.73	-	-	-
Marital status	1.12	0.64—1.94	0.68	-	-	-
Education level	1.44	0.78—2.80	0.25	-	-	-
Duration since treatment	0.95	0.88—1.01	0.12	-	-	-
Personal health	1.24	0.18—5.29	0.78	-	-	-
Sex worker	2.52	0.75—7.58	0.10	2.44	0.70—7.75	0.13
MSM	1.5	0.06—1.63	0.74	-	-	-
Past blood transfusion	0.62	0.31—1.15	0.14	0.47	0.11—1.63	0.26
Circumcision	0.82	0.16—6.01	0.82	-	-	-
History of STIs	1.47	0.65—3.08	0.32	-	-	-
Multiple sex partners before HIV diagnosis	**1.68**	**0.97—2.92**	**0.06**	2.73	0.84—9.50	0.09
Condom use	1.41	0.68—3.22	0.37	-	-	-
IDUs	2.18	0.10—2.31	0.52	-	-	-
Surgical history	0.61	0.032—3.52	0.64	-	-	-
CD4 count cells	1	0.99—1	0.32	-	-	-

Significant results are given in bold.

OR: odd ratio; CI: confidence interval; MSM: men who have sex with men; IDUs: intravenous drug users; STI: sexually transmitted infection.

## Discussion

Congo is a highly informative country to study HHV-8 infection due to a significant proportion of immunosuppressive agents such as HIV, also of its geographic location in an area described as the KS belt^12,22^. This study is first to investigate the seroprevalence and molecular diversity of HHV-8 in the Congolese population by considering their HIV-1 serostatus.

This study reported HHV-8 seroprevalence of 19% among PLWH in Brazzaville, Congo. Our results is in agreement with the low prevalence (12%) found in HIV positive prostitutes in Kenya^23^. By contrast, this result was not consistent with Congo seroprevalence estimation reported by Cesarman et al. which would be between 41 and 70%^24^, and is threefold lower than HHV-8 seroprevalence reported in three studies carried out in 2013, 2014 and 2015 in Cameroon among people living with HIV, which was 61%, 98% and 79% respectively^11,25,26^. Seroprevalence rates higher than 70% in the context of HIV have been also reported in Tanzania and Nigeria^27,28^. On the other hand, Gabon, Central African Republic and Democratic Republic of Congo which are neighbouring countries, conducted several studies in non-HIV patients and found that HHV-8 seroprevalence rate was 35%, 94% and 82%, respectively^29–31^. The low HHV-8 seroprevalence in our study compared to other countries can be explained by the various degree of immunosuppression of patients between studies, which has an impact on the techniques used to detect HHV-8 antibodies to the point of underestimating the HHV-8 seroprevalence in immunocompromised populations. In other studies conducted in the sub-region, the lytic antigen ELISA assay was the most used which can overestimate HHV-8 seroprevalence, in particular because of EBV-cross-reactive antibodies. Moreover, the low seroprevalence of HHV-8 found in Brazzaville corroborates the prevalence of Kaposi’s sarcoma which is 0.2% according to the Brazzaville cancer register^32^ while in Cameroon the high prevalence of HHV-8 is correlated with that of Kaposi's sarcoma^26,33^. The strong representation of women among the participants is explained by the fact that HIV is more prevalent in women than men in Congo, as previously reported in other studies^9,34,35^. However, here men were more frequently HHV-8 infected than women, in line with the sexual route of HHV-8 transmission, according to studies^36,37^. Furthermore in other studies of the sub region on KS, men were more prone to the development of KS than women; almost twice to thrice more affected^38–40^. Although MSM population was weakly represented in our study, a trend was observed in patients who had multiple sexual partners before HIV diagnosis, leans in favor of heterosexual transmission in our study group. Ugandan, Kenyan and Iranian studies suggest heterosexual transmission of HHV-8 respectively, in patients who reported having multiple marital unions, sex workers and Iraqi blood donors who reported frequent legal and illegal sex^23,34,36^. Other studies indicate that, in KS belt areas and some countries, the transmission is probably mainly sexual with a risk of infection correlating with the number of sex partners^9,41,42^. We did not identify any signal of parenteral transmission in the present study, as no difference of HHV-8 seroprevalence was evidenced in the two groups for IDU patients, transfusion or circumcision.

Furthermore, to know HHV-8 subtypes circulating in Brazzaville, we analyzed the sequences of the ORF K1 region of patients with detectable HHV-8 DNA but asymptomatic to HHV-8 associated diseases. Among them, 6 samples were typable, and subtype A5 was the most predominant (50%), followed by B1 variant of subtype B (33%) and a subtype C variant close to C7 (17%). A5 and B subtypes are the most frequent subtypes found in sub-Saharan Africa, with a distribution on the African continent of 42%, 27% and 18% for subtypes B, A5 and C respectively^16,43,44^. Our results are consistent with *Betsem *et al*.* which finds subtype A5 and B1 variant of subtype B circulating mainly in the Cameroonian population^33^. Of note, A5 and B subtypes are also actively circulating among KS patients in the Central African Republic^43,45^; in Zimbabwe, South Africa and Uganda^16,19,46^. In contrast, *Lacoste *et al*.* in a sub-regional study including a Congolese patient with MCD, described subtype B as the only one circulating in Congo. *Varmazyar *et al*.* reports that subtype A was detected more frequently among HIV-infected patients with or without KS than HIV-negative subjects^15^. Previous studies have demonstrated the clustering patterns of HHV-8 subtypes with geography and ethnicity, and these may have arisen through ancient human migrations^44,47^. In addition, *de Oliveira Lopes *et al*.* after analysis of more than a hundred sequences of subtype B from different regions of the world, shows that the HHV-8 subtype B circulating in Congo is one of the oldest; and it is possible that this subtype was brought by African slaves during the colonial period of Brazil^48^. Interestingly, we identified a subtype C variant close to C7 found in Moroccan and Central African populations^43,45,49^, while subtype C occurs predominantly in Europe, USA, the Middle East, the Mediterranean, and Asia^20^. The discovery of subtype C in our study may be explained by human migrations. Our study may have some limitations. The patients in our study were on antiretroviral treatment but did not benefit from regular biological monitoring, which unfortunately did not allow us to get the CD4 cells count and the HIV viral load for each patient. In addition, the sample size studied is limited and probably explain why statistical results did not rich significancy.

In conclusion, we observed a relatively low seroprevalence of HHV-8 in PLWH, with a more frequent carriage in men, asymptomatic oral excretion and a predominance of subtype A5. A larger study, including non-HIV participants is needed to determine global seroprevalence, and to know if other subtypes circulate in Congo, as shown by the presence of a subtype C in Brazzaville.

## Materials and methods

### Study population

This prospective cross-sectional study was conducted between July and October 2019 in seven health facilities following more than 5890 patients living with HIV in Brazzaville, Congo. In total, 353 patients under highly active antiretroviral therapy (HAART), 18 years of age or older, HIV-1 positive and with signed written consent were included.

### Data and sample collection

For each patient, a survey questionnaire was completed with data on socio-demographic characteristics including gender, age, marital status and education level. Information on HIV infection, including clinical stage at inclusion, HAART regimen in accordance with the guidelines of the Congo and date of initiation, CD4 count cells, as well as information’s on sexual behaviour (number of different partners, condoms using, homosexuality, history of sexually transmitted infection (STI)) were collected.

For each patient, whole blood, serum and oral dry swab were collected. Within the following hour, the samples were transferred to the Brazzaville University Hospital Laboratory and then stored at -80 °C. All samples were coded by a unique identification number assigned to each participant and checked by a technician external to the study on compliance before shipment. Finally, the samples were sent in the virology department of the Pitié-Salpêtrière Hospital (UMR Sorbonne Université INSERM 1136) to perform HHV-8 serological and molecular assays.

### HHV-8 serology

Sera were tested for HHV-8 antibodies directed against LANA-1 IgG (latency associated nuclear antigen) by an indirect immunofluorescence assay using the BC- 3 cell line infected with HHV-8 but not with Epstein Barr virus, as described previously^50^. This technique, using unstimulated cells, has a sensitivity between 80 and 85% and a very good specificity (nearly 100%). Note that, the term “equivocal HHV-8 serology results” was used when after several tests in some patients, we had an indeterminate result, i.e. ambiguous, fluorescent slide on reading.

### HHV-8 molecular assay

DNA extraction from whole blood and oral swab discharged in PBS (200 microL) of patients with positive HHV-8 serology were performed using the NucliSens® EasyMAG kit (BIOMERIEUX SA, FR) according to manufacturer’s instructions.

#### Detection and quantification of HHV-8 DNA

The extracted DNA was used for the detection and the quantification of HHV-8 in blood and saliva. By real-time PCR, primers (forward KS1: 5’-CCGAGGACGAAATGGAAGTG-3’ and reverse KS 2: 5’-GGTGATGTTCTGAGTACATAGCGG-3’) and probe [5’-(6FAM) ACAAATTGCCAGTAGCCCACCAGGAGA (TAMRA)-3’], designed on the ORF73 gene encoding LANA-1 protein were used to detect and to quantify HHV-8 DNA, as previously described^51^.

#### Sanger sequencing of ORF-K1 and typing

A 679 base pair (bp) fragment of ORF-K1 including the 2 hypervariable regions, was amplified by nested PCR, as previously described^20^. After multiple alignment of nucleotide ORF-K1 sequences (including reference sequences collected on NCBI database) with Mafft7, phylogenetic analysis was performed with PhyML3.0, GTR model and 1000 bootstraps resampling.

### Statistical analysis

Field and laboratory data were entered in Excel version 2016. Continuous variables were expressed as medians with interquartile ranges [IQR], and discrete variables as numbers and percentages.

GraphPad software was used to perform nonparametric tests, Mann–Whitney U tests for quantitative data, Fisher exact t or Chi2 square tests for qualitative data and *p* < *0.05* was considered significant. Univariable and multivariable (including gender, age, education level, sex workers, men who have sex with men (MSM), blood transfusion, circumcision, history of STIs, multiple sex partners before HIV diagnosis, unprotected sex, intravenous drug users (IDUs), history of surgery and CD4 count cells) logistic regression analyses were performed to identify risk factors associated with HHV-8 infection. Factors with a *P value* < 0.20 in the univariate logistic regression analyses were included in the multiple logistic regression model.

#### Ethics statement

Approvals were obtained from the Health Sciences Research Ethics Committee (reference number: 222 / MRSIT / IRSSA / CERSSA) which is the highest approval institution for human experiments, this one is under the supervision of the Ministry of Scientific Research of the Republic of Congo. The study then received secondary authorization from the Faculty of Health Sciences of the University Marien NGOUABI (reference number: 74 / UMNG.FSSAV-DOY). All experiments were performed in accordance with relevant named guidelines and regulations. Voluntary participation, confidential results and anonymous data processing. Written informed consent was obtained from all participants.

## Supplementary Information

Supplementary Tables.
