# Supplementary material for: Seroprevalence and molecular diversity of Human Herpesvirus 8 among people living with HIV in Brazzaville, Congo
Source: Sci Rep. 2021 Aug 31;11:17442. doi: 10.1038/s41598-021-97070-4 (PMC8408137; doi:10.1038/s41598-021-97070-4)
Supplement: Supplementary file 1 — Supplementary Tables. [file 41598_2021_97070_MOESM1_ESM.docx]

| **Table S1**: Characteristic of participants who have equivocal HHV-8 results | |
| --- | --- |
| Variable | Equivocal HHV-8 results (N=12) |
|  | No. (%) |
| Male | 2 (16.67) |
| Female | 10 (83.33) |
| Congolese nationality | 12 (100) |
| Age, median (IQR), years | 43.5 (40.5-54) |
| Duration of HIV treatment, median (IQR), years | 4 (0.75-7.25) |
| Single | 6 (50) |
| Married | 2 (16.67) |
| Widower | 2 (16.67) |
| Common-law union | 1 (8.33) |
| Divorced | 1 (8.33) |
| Uneducated | 0 |
| Primary | 6 (50) |
| Secondary (I & II) | 4 (33.33) |
| Superior | 2 (16.67) |
| Early HIV (Stage 1&2) stage at initiation | 2 (16.67) |
| Late HIV (Stage 3 & 4) stage at initiation | 3 (25) |
| Not specified stage at initiation | 7 (58.33) |
| First line treatment | 12 (100) |
| Second line treatment | 0 |
| Non-medical personnel | 11 (91.67) |
| Medical personnel (Nurse) | 1 (8.33) |
| Sex Worker | 0 |
| MSM | 1 (8.33) |
| Past blood transfusion | 3 (25) |
| Circumcision | 2 (16.67) |
| IDUs | 0 |
| History of STIs | 0 |
| Multiple sex partners before HIV diagnosis | 7 (58.33) |
| Condom use | 2 (16.67) |
| Unsafe sex practices | 3 (25) |
| Surgical history | 1(8.33) |
| IQR: interquartile; MSM: men who have sex with men; IDUs: intravenous drug users; STI: sexually transmitted infection; HIV: human immunodeficiency virus. | |

| **Table S2**: Characteristics of patients with detectable and undetectable viral loads | | | | |
| --- | --- | --- | --- | --- |
| Variable | Patients with detectable viral load  (N=13) | | Patients with undetectable viral load  (N=51) |  |
|  | No. (%) | | No. (%) | P value |
| Male | 5 (38.46) | | 19 (37.25) | 0.93 |
| Female | 8 (61.54) | | 32 (62.75) |  |
| Congolese nationality | 13 (100) | | 47 (92.15) | NC |
| Other nationality | 0 | | 4 (7.85) |  |
| Age, median (IQR), years | 45 (30-53) | | 45 (35-54.5) | 0.66 |
| Duration of HIV treatment, median (IQR), years | 3 (1-4) | | 5 (1-8) | 0.64 |
| CD4 count, median (IQR), Cell/µl | 373.5 (195.8-550.8) | | 478.5 (121-1744) | ˂0.0001 |
| Single | 9 (69.23) | | 19 (37.25) | NC |
| Married | 2 (15.39) | | 13 (25.5) |  |
| Widower | 0 | | 6 (11.76) |  |
| Common-law union | 1 (7.69) | | 8 (15.69) |  |
| Divorced | 1 (7.69) | | 5 (9.8) |  |
| Uneducated | 0 | | 1 (1.96) | NC |
| Primary | 4 (30.77) | | 10 (19.6) |  |
| Secondary (I & II) | 7 (53.85) | | 25 (49.02) |  |
| Superior | 2 (15.38) | | 15 (29.42) |  |
| Early HIV (Stage 1&2) stage at initiation | 3 (23.075) | | 10 (19.6) | NC |
| Late HIV (Stage 3 & 4) stage at initiation | 3 (23.075) | | 6 (11.77) |  |
| Not specified stage at initiation | 7 (53.85) | | 35 (68.63) |  |
| First line treatment | 12 (92.31) | | 49 (96.08) | 0.5 |
| Second line treatment | 1 (7.69) | | 2 (3.92) |  |
| Non-medical personnel | 13 (100) | | 49 (96.08) | NC |
| Medical personnel (Midwife, Red cross staff) | | 0 | 2 (3.92) |  |
| Sex worker | 0 | | 5 (9.8) | NC |
| MSM | 0 | | 1 (1.96) | NC |
| Past blood transfusion | 4 (30.77) | | 10 (19.6) | 0.87 |
| Circumcision | 4 (30.77) | | 18 (35.29) | 0.6 |
| IDUs | 0 | | 1 (1.96) | NC |
| History of STIs | 2 (15.38) | | 8 (15.69) | 0.72 |
| Multiple sex partners before HIV diagnosis | 6 (46.15) | | 30 (58.82) | 0.53 |
| Condom use | 2 (15.38) | | 7 (13.72) | 0.85 |
| Unsafe sex practices | 2 (15.38) | | 22 (43.13) | 0.19 |
| Surgical history | 1 (7.69) | | 0 | NC |
| IQR: interquartile; MSM: men who have sex with men; IDUs: intravenous drug users; STI: sexually transmitted infection; HIV: human immunodeficiency virus; NC: non calculated. | | | | |
|  | | | | |
